# A systematic review of published interventions for primary and secondary prevention of ischaemic heart disease (IHD) in rural populations of Australia

**DOI:** 10.1186/s12889-016-3548-1

**Published:** 2016-08-27

**Authors:** Laura V. Alston, Karen L. Peterson, Jane P. Jacobs, Steven Allender, Melanie Nichols

**Affiliations:** WHO Collaborating Centre for Obesity Prevention, Faculty of Health, Deakin University, Locked Bag 20001, Geelong, VIC 3220 Australia

**Keywords:** Rural, Australia, Ischaemic heart disease, Inequalities, Intervention

## Abstract

**Background:**

Rural Australians are known to experience a higher burden of ischaemic heart disease (IHD) than their metropolitan counterparts and the reasons for this appear to be highly complex and not well understood. It is not clear what interventions and prevention efforts have occurred specifically in rural Australia in terms of IHD. A summary of this evidence could have implications for future action and research in improving the health of rural communities. The aim of this study was to review all published interventions conducted in rural Australia that were aimed at the primary and/or secondary prevention of ischaemic heart disease (IHD) in adults.

**Methods:**

Systematic review of the peer-reviewed literature published between January 1990 and December 2015. Search terms were derived from four major topics: (1) rural; (2) ischaemic heart disease; (3) Australia and; (4) intervention/prevention. Terms were adapted for six databases and three independent researchers screened results. Studies were included if the published work described an intervention focussed on the prevention or reduction of IHD or risk factors, specifically in a rural population of Australia, with outcomes specific to participants including, but not limited to, changes in diet, exercise, cholesterol or blood pressure levels.

**Results:**

Of 791 papers identified in the search, seven studies met the inclusion criteria, and one further study was retrieved from searching reference lists of screened abstracts. Typically, excluded studies focused on cardiovascular diseases without specific reference to IHD, or presented intervention results without stratification by rurality. Larger trials that included metropolitan residents without stratification were excluded due to differences in the specific needs, characteristics and health service access challenges of rural populations. Six interventions were primary prevention studies, one was secondary prevention only and one included both primary and secondary intervention strategies. Two interventions were focussed exclusively on Aboriginal and Torres Strait Islander (Australian Indigenous) populations.

**Conclusions:**

Few interventions were identified that exclusively focussed on IHD prevention in rural communities, despite these populations being at increased risk of IHD in Australia, and this is consistent with comparable countries, internationally. Although limited, available evidence shows that primary and secondary interventions targeted at IHD and related risk factors can be effective in a rural setting.

**Electronic supplementary material:**

The online version of this article (doi:10.1186/s12889-016-3548-1) contains supplementary material, which is available to authorized users.

## Background

Globally, more people die from cardiovascular diseases (CVD), than any other cause [1]. In 2013, 29.5 % of Australian deaths were attributed to CVD, making it the most common cause of death [2]. ischaemic heart disease (IHD) is the most prevalent CVD and is defined clinically as acute myocardial infarction (AMI) or angina pectoris [2], and it is estimated that, globally, 7.4 million people die from IHD each year [1]. IHD has been the leading single cause of death in Australia since 2000 [2]. These conditions appear to affect some populations more than others [3], particularly those living in rural areas, people of Aboriginal and Torres Strait Islander (ATSI) heritage and people of lower socio-economic status (SES) [4, 5]. Modelled estimates of Australian mortality figures between 2009 and 2011 suggest that more than 1200 lives would have been saved annually, if people living in rural areas had the same IHD mortality rate as metropolitan counterparts [6]. Overall IHD mortality rates in Australia decreased substantially between 2001 and 2010, though these decreases were smaller in more remote areas than in major cities (−4.1 % for males and −4.3 % for females in major cities, compared to −2.4 % and −3.9 % in remote areas) [5].

The increased burden of IHD in rural areas of Australia, despite overall mortality decline, is comparable to patterns observed in high income countries internationally including in rural Scotland, Norway and the United States (US) [7–9]. Decreases in IHD have been observed in high-income countries, such as in the US and UK, and have been largely attributed to primary and secondary prevention efforts that have led to the reduction in modifiable risk factors such as hypertension, cholesterol and smoking, as well as advances in medical therapies [9–11].

Risk factors for IHD are interlinked, with modifiable factors including tobacco smoking, poor nutrition, physical inactivity, obesity, high blood pressure and high blood cholesterol [5]. These risk factors are also common to other major non-communicable diseases (NCDs), including stroke, cancer, respiratory disease and diabetes [12]. The importance of these risk factors is emphasised by the World Health Organisation’s 25x 25 goal, which identifies them as significant targets to achieve the goal of reducing premature mortality from NCDs by 25 % by the year 2025 [12, 13].

CVD, including IHD and stroke, has been identified as a high priority in rural Australia with particular reference to primary prevention strategies focused on improving nutrition and physical activity and reducing tobacco smoking [14]. The disparity between rural and metropolitan mortality and disease rates represents an important equity target for any prevention strategy and there is some limited evidence internationally for the effectiveness of community level prevention efforts in rural communities, when they are tailored specifically to the needs of the target population [11, 15]. Interventions attempting to address the increased burden of IHD in rural areas need to take into account the ways in which rural and metropolitan populations differ, which include health care access, education, income and risk factor prevalence. The aim of this study was to systematically review all published literature since 1990 reporting interventions that focussed on reducing the IHD burden in rural Australia, through primary or secondary prevention, and to synthesise the available evidence on the efficacy of such prevention efforts.

## Methods

### Data sources

We sought to identify studies within the published peer-review literature that were focussed on rural populations and that aimed to prevent or reduce IHD burden or risk factors. This systematic review was registered with Prospero, (number CRD42016033431). The term ‘rural’ used throughout this paper, refers to all areas classified as being outside of major cities of Australia, by the Australian Bureau of Statistics, Accessibility Remoteness Index of Australia (ARIA) [16]. ARIA has five categories of remoteness, which are, in increasing order of remoteness major cities, inner regional, outer regional, remote and very remote. These definitions are based on remoteness scores derived from relative road distance to population localities and services [16].

Search terms used were related to four major topics including, ‘ischaemic heart disease’, ‘rural’, ‘intervention or prevention,’ and ‘Australia’. The search was conducted in November and December of 2015. The six databases included in the search were CINAHL, Medline, Academic Search complete, Rural and Remote Health Database, Health and Society Database and Embase. An additional hand search was undertaken of reference lists from included studies.

The following were the Inclusion criteria:

- Studies had to be published in peer review journals from 1990 to 2015.
- Population: The study had to be focussed on a population of adults living exclusively in a rural area of Australia. Larger trials that included both rural and metropolitan residents without stratification by rurality were excluded.
- Intervention: Interventions reporting an explicit aim of primary or secondary prevention of heart disease, with specific mention of IHD as a target. For example, if a study referred only to CVD as a whole, and not specifically to IHD, it was excluded.
- Comparator: Comparisons between intervention groups and control group (preferably), or relevant health survey data or baseline results. Comparison to a non-rural population was not necessary for inclusion.
- Outcomes: Including but not limited to: changes in behavioural risk factors (including exercise, diet, alcohol, smoking and stress management), knowledge of heart disease, health assessment measures (e.g. blood pressure, cholesterol, blood glucose levels, obesity or weight), and rates of mortality, morbidity, case fatality, hospital admissions, or complications.
- Study design: All types of intervention designs were considered in this review. Studies describing intervention models (study design/protocol papers) that did not present intervention results were excluded.

### Study selection, data extraction and analysis

**Fig. 1:**
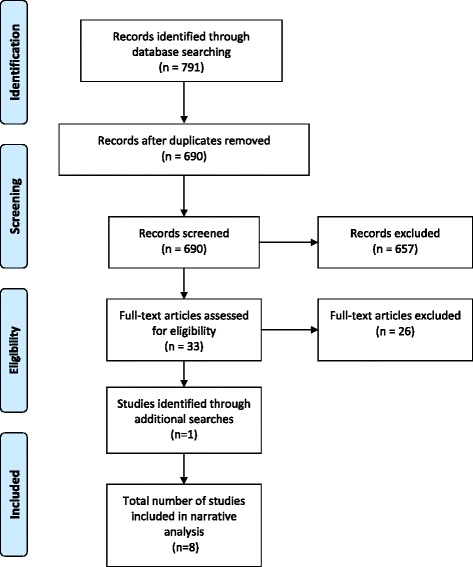
PRISMA diagram of the systematic review process for this review

The lead researcher (LA) reviewed all results from the six databases, removed duplicates and screened all results based on titles and, abstracts against the review criteria (see Fig. 1). Two additional researchers (KP & JJ) each screened a 50 % sample of titles and abstracts as a second reviewer. Any discrepancies were identified and resolved by consensus among the three researchers producing a list of papers for full text assessment for eligibility against the review criteria. Reference lists of all full texts were then searched for additional potentially eligible studies.

Data were extracted into a spreadsheet from the full texts by the lead researcher. The details collected included the publication details of the study, years of intervention, intervention type, follow up period, outcome measures (such as changes in clinical and modifiable risk factors), results and authors’ conclusions. Each intervention was then then categorised as either primary or secondary prevention, or both, and by the broad type of intervention (e.g. delivery through initial screening/education/exercise or whole community programs). The studies were then synthesized into a narrative analysis, with a focus on changes in outcome measures. We applied a narrative analysis because quantitative meta-analysis was deemed inappropriate due to the small sample size and heterogeneity of the interventions returned by the search strategy.

### Quality analysis

Two researchers independently assessed each study using the Cochrane Collaboration’s tool for assessing risk of bias [16]. The tool is used to assess the risk of bias within each individual study based on five different types of bias including: selection bias (randomisation of participants), performance bias (blinding of participants), detection bias (blinding of outcome measures), attrition bias (incomplete outcome data) and complete reporting. Studies were rated as either high, low or unclear risk against each of the criteria. The Cochrane tool does not use a total score to assess overall risk of bias, so each type of bias is assessed individually.

## Results

Of the initial 791 papers returned by the database search, 33 full texts were screened, and of these, seven studies met the inclusion criteria. Major reasons for exclusion at full text stage included that the study did not specifically refer to IHD, (usually only reporting on CVD as a whole). One further eligible peer reviewed study was identified through hand searching of reference lists, resulting in a total of eight studies included in the review. Details of interventions, outcome measures, results and conclusions of the studies are described in Table 1. Across these eight studies, five were conducted in ‘inner regional areas’ [17–21], two in ‘outer regional areas’ [22, 23] and one in a ‘very remote’ area [24]. No studies included here were conducted in remote areas of Australia. Two included interventions focussed exclusively on Aboriginal and Torres Strait populations [22, 24], four included a screening component part of the intervention [18, 20–22] one evaluated the effectiveness of a long term, whole community intervention [19] and one study included an assessment of cardiac rehabilitation [17].

**Table 1:** Characteristics of prevention programs aimed at reducing ischaemic heart disease burden in rural Australia

Author, year of publication	Year(s) of study	Intervention strategies	Participants, follow up	Outcome measures	Results	Conclusions
Aoun & Rosenberg, 2004 [17]	2000–2001	7 week cardiac rehabilitation program	*N* = 203 patients with current CVD diagnosis, *n* = 159 controls. Followed up at post program, 3, 6 and 12 months	Self-reported changes in:		Cardiac Rehab programs in rural areas are successful in reducing risk factors for IHD and improving quality of life
				-WT	-WT: ↓ 0.5 kg	
				-PA (6 min walk test)	(*p* = 0.004)	
				-BP,	-PA: 431.6 m to 469.6 m (*p* < 0.001)	
				-Quality of life scores (QoL)	-BP: NS, *p* value not reported	
					-QoL: 80.69 (15.9) to control 71.6 (18.86) (*p* = 0.04)	
Burgess et al., 2015 [22]	2012–2014	Cardiac prevention screening services within primary health teams	Aboriginal clients aged 20 years and over, *N* = 2586 identified as high risk. Followed up every 3 months for two years	Achievement of target (not compared to baseline for significance):	Achieved target post program:	This type of program is a feasible way of reducing IHD risk factors in rural indigenous populations
				-BP	-BP: 57 %	
				-TC	-TC : 40 %	
			No control group	-% Stopped smoking	-Stopped smoking: 50 %	
Carrington and Stewart, 2015 [18]	2009–2010	Nurse-led screening and education program	*N* = 530, pre/post follow up design, no control group. Followed up at 6 months	Mean change in	-BP diastolic: ↓ 4 mmHg Systolic: ↓ 1 mmHg	Feasibility of a nurse-led screening and intervention was shown for a rural population
				-BP		
				-TC		
				-WT (kg)	-TC: ↓ 0.6 mmol/L	
				-BMI		
					-WT: ↓ 1.0 kg	
					-BMI: ↓ 0.3mkg^2^	
Higginbotham et al., 1999 [19]	1980–1990s (exact years not specified)	Whole community intervention	*N* = 359, no control group, but rates compared to nearby region	Change in	Intervention area:	Whole community interventions can have multiple positive impacts in rural communities and possibly reduce IHD burden if implemented with consideration of community needs and subgroups
				-IHD Mortality (age standardised rates (per 100,000))	Women (35-64y)	
					Fatal MI: −14.2 (95 % CI: −26.0, −2.4)	
			9 year data collection phase	-Non-fatal MI rates,	Non-fatal MI: 1.7 (95 % CI: −4.4, 7.9)	
				-Case fatality compared to non-intervention region	Men (35-64y)	
					Fatal MI: −10.9 (95 % CI: −18.2, −3.6)	
					Non-fatal MI: 3.2 (95 % CI: −0.6, 7.0)	
					Rates declined faster in intervention population compared to than non-intervention region	
Krass et al., 2003 [20]	Year(s) of intervention not specified	Pharmacy screening and education program	*N* = 389 adults in regional area, followed up from baseline to 3 months, no control group	From baseline to 3 months:	% Inactive	Community Pharmacies have the potential to increase resource provision in rural areas and can be effective at reducing risk factors for IHD
					Cohort 1	
				Change in	57 % to 44 % (*p* < 0.0001) Cohort 2	
				-BP		
				-TC		
				-% Current smokers	50 % to 44 % (*p* = 0.01)	
				-% Not meeting PA recommendations	% Smokers = No change	
				-% Of people by BMI category	Both Cohorts:	
					Mean TC: ↓ 0.26 mmol/L (95 % CI 10–0.42) (*p* < 0.003).	
					BP: ↓ 10.5 mmHg (95 % CI 4.0-16.9) in mean systolic BP within Cohort 1 (*p* = 0.012), no difference for cohort 2.	
					BMI = NS (*p* value NR)	
Kerr et al., 2008 [23]	Year(s) of intervention not specified	Exercise and cardiovascular monitoring program	*N* = 164 war veterans, followed up at 3, 6, 12 months	3 monthly follow up:	12 months:	This type of program was shown to be effective at reducing risk factors in a high risk, regional population of males
				-Diastolic and systolic BP (mmHg)	Resting HR:↓ 4.0 bmp	
				- HR (bpm)	Diastolic BP: ↓ 6.4 mmHg	
					Systolic BP: ↓ 8.4 mmHg (*p* = <0.05). Weight (kg) :NS	
Ray, 2001 [21]	Year(s) of intervention not specified	Once-off mobile heart screening program	*N* = 135 adults aged 30–69 years followed p 6 months post intervention	Self-report change in health behaviour after screening	Self-report health behaviours:	Heart risk screening can be a motivator for health behaviour change
					76 = positive change	
					59 = no change	
Rowley et al., 2000 [24]	1993–1995	Lifestyle education program	Aboriginal community participants	Change in risk factors overtime (Intervention group either compared BL or to control):	-no significant change in dietary and physical activity when compared to controls.	Some short term changes were not sustained in metabolic profiles from this intervention, however this program was found to be sustainable for this type of rural community
			*N* = 32 intervention,			
			*N* = 17 controls			
			followed up at, 6 months, 2 years	-BMI		
				-Fasting glucose	-BMI: ↓from BL at 6 months (to control: *p* = 0.012), 12 months: NS (*p* = NR)	
					-Fasting glucose:	Positive changes in awareness and behavioural risk factors were noted
					6 months:↓ 0.9 mmol (intervention to baseline *p* = 0.021)	
				- Glucose tolerance (oral glucose tolerance test (OGTT))	Intervention to control : NS (*p* = 0.132)	
					−2 h post -OGTT:	
				-plasma insulin	6 months: ↓ 1.6 mmol/l (*p* = 0.01 to BL)	
				-triglyceride concentration		
					Intervention to control: NS *p* = 0.154	
					-Fasting insulin: Intervention to control NS (*p* = 0.103)	
					-Fasting triglycerides: NS (*p* = 0.158)	

*Abbreviations*: *BL* baseline, *BMI* body mass index, *BP* blood pressure, *HR* heart rate, *bpm* beats per minute, *IHD* ischaemic heart disease, *MI* myocardial infarction, *NS* not significant, *NR* Not reported, *OGTT* oral glucose tolerance test, *PA* physical activity, *TC* total cholesterol, *QoL* quality of life, *WT* weight (kg), ↓: decrease

### Primary prevention

Six primary prevention studies were identified; five in inner and outer regional areas [18, 20–23], and one in a remote area [24]. Two reported on interventions in ATSI populations [22, 24]. Most studies [17, 20, 21, 24] were published more than 8 years ago. The intervention activities included an exercise program for a high risk population [23], cardiac rehabilitation [17], a full community intervention [19], and five risk factor screening and/or subsequent education or treatment programs within small communities [18, 20–22, 24]. Generally, the studies reviewed showed that IHD prevention efforts in rural communities are feasible and were effective in either reducing one or more risk factors, or IHD mortality, however the studies were limited by short follow up periods, small population numbers and a lack of inclusion of control groups in study designs.

Kerr et al. [23] measured the effect of a 12-month exercise program on IHD risk factors in a population of war veterans living in regional Queensland (*n* = 164), without a control group. The main outcome measures included measurement of heart rate (HR), blood pressure (BP), skinfold and girth measurements, exercise heart rate response and estimated aerobic capacity. The results were used to determine if the program could be effective in this rural, high risk population. The study showed that an organised exercise group could be feasible in a rural setting for high risk clients, with positive effects shown for resting HR (−4 bpm), diastolic (−6.4 mmHg) and systolic BP (−8.4 mmHg) by the end of the program (*p* = <0.05). Weight was unchanged at 12 months, however there were some improvements in body composition. The generalisability of results from this study is limited because only 54 % of participants completed the final 12 month follow up assessment.

Three studies [18, 20, 21] used primary risk factor screening as the start point of the intervention, screening patients’ BP, cholesterol and BMI, and when compared to baseline, showed that these types of programs are potentially feasible in rural areas. Krass and colleagues [20], assessed the impact of a pharmacy screening and health promotion program (*n* = 389) on the risk of IHD and stroke in two towns in regional New South Wales. The health promotion program included individual education on lifestyle improvements including diet, exercise and smoking cessation advice. After three months, significant changes were observed in mean total cholesterol for both towns (−0.26 mmol/l, 95 % CI 0.10-0.42, *p* = <0.003), while BP was reduced in participants from one town (−10.5 mmHg, 95 % CI 4.0-16.9, *p* = 0.012), changes in physical activity and smoking prevalence were reported, with increases in activity reported by participants at 3 months. The authors noted that there was little change in smoking prevalence, and attributed this to the relatively short period of intervention and follow up of this study design, which with no control group comparison, would also make it difficult to draw concrete conclusions on the overall effectiveness of this intervention. Carrington and Stewart [18], describe a similar, yet nurse-led intervention in regional Victoria in which 530 self-selecting patients were screened for cardiovascular risk, then provided with counselling and advice tailored to their risk level [18]. Just over 60 % of the patients (*n* = 326) had clinically significant improvements in risk factor levels at 6 months post-intervention, with BP, total cholesterol and weight all decreasing from baseline levels, yet these results were not compared to a control group. No further follow up was undertaken after 6 months post-intervention, making it difficult to determine either the sustainability of this design or the long term impact on risk factors in the rural community studied. A third screening study published in 2001 [21], without inclusion of controls, assessed the effect of a one-time screening session delivered by the mobile ‘Heart Bus’ on self-reported behaviour changes in inner regional Queensland. The study followed up 135 participants and found that 76 % of participants self-reported they had made positive changes to their diet and exercise behaviours 6 months later. The major limitations of this study include the small sample design, lack of control and the self-reported nature of all of the outcome measures.

Two studies assessed IHD interventions in ATSI populations only [22, 24] and showed that IHD primary prevention efforts in these populations have the potential to be effective. One study was a clinical audit of cardiac prevention and screening services [22], and the other was an assessment of a diet and exercise program with comparison made to a self-selected control group [24]. Burgess et al. [22] analysed the effectiveness of cardiac prevention services through clinical audits every three months of cardiovascular risk assessments, and level of pharmaceutical prescription delivered through primary health care services over 2 years. The study focussed on results from 2586 participants, who were identified to have a five year CVD risk of 16 % or greater. Blood pressure medication was prescribed for 67 % of participants and lipid lowering medications for 55 %, with clinical follow-up every three months to assess if target levels were achieved. By the end of the two year evaluation, the number of participants who achieved clinical targets for BP was 1366 (56 %) while 989 (40 %) reached targets for cholesterol, however changes in the proportion of participants reaching targets did not change significantly over time for either outcome. Rowley and colleagues [24] assessed the effectiveness of a primary health care service providing diet and/or exercise education and support in a small rural ATSI population. The study included an intervention group (*n* = 32) and a self-selected control group (*n* = 17). There were significant differences (*p* = 0.03) observed between the intervention and control arms, for mean change in 2 h plasma glucose, and triglyceride levels at two years post-intervention. There were also changes in dietary behaviour and physical activity in the intervention group, however these did not appear to be significant when compared to controls. Response rates ast the two year follow up were low for younger participants aged 15–34, with 43 % responding, compared to 80 % for those aged 35 and over, however results shown here are clearly limited by the small study sample.

### Secondary prevention

Only one secondary prevention intervention [17] was identified. The study evaluated a 7-week bi-weekly education and exercise cardiac rehabilitation program in a rural area of Western Australia (Heart Smart), and included 203 participants with a current CVD diagnosis. The evaluation compared quality of life and cardiac knowledge scores of these participants with to 159 non-participants (who were eligible, but did not wish to participate in the intervention). All follow up data, including clinical measures were self-reported. After 6 months, the intervention group had reportedly increased their physical activity (*p* < =0.001), and reduced their weight (−0.5 kg, *p* < 0.05) from baseline. Higher quality of life and cardiac knowledge scores were observed for the intervention group, however the non-intervention group had a low response rate to the follow up survey (42 %). Self-reported cholesterol levels were 3.6 mmol/L at baseline, and these reduced to 2.8 mmol/L by 6 months post follow up, however no changes were observed for self-reported BP. The authors did not specify if these measures were taken by the same medical clinic, or if any clinical documentation was collected with self-report results.

### Primary and secondary prevention

Higginbotham et al. [19] was the only study to include both a primary and secondary prevention program. The Coalfields Healthy Heartbeat was a 10-year community intervention, which employed multiple strategies and included health promotion and awareness advertising, mobilisation of community resources, school health-promotion programs, exercise, cooking and education groups, and a cardiac rehabilitation program. The remainder of the Hunter Valley region served as a comparison population for the program evaluation. The authors found a larger reduction in fatal AMI cases in the intervention area relative to the comparison population over a 9-year monitoring period [19], however there was no reduction in non-fatal AMI rates. There were no significant differences in risk factors changes between the intervention and non-intervention areas.

Table 1: Characteristics of prevention programs aimed at reducing IHD burden in rural Australia.

### Quality assessment

Due to the lack of randomization used in the design of all studies and minimal use of control groups, all of the included studies were assessed to have a high risk of bias for the selection, performance and detection criteria of the Cochrane tool, and low or unclear risk for attrition and reporting bias criteria. Only one study reported using a behavioural science theory in the design of their study, identifying the health promotion and behavioural change models in the design of their follow up questionnaire [16]. There was also a lack of detail around the length of the intervention, and follow up periods for 4 of the 8 studies, and generally follow up periods were small.

## Discussion

Eight studies were identified that met the inclusion criteria, indicating that there is little published work about IHD prevention efforts occurring in rural communities in Australia. All studies based in inner and outer regional areas [16], found that primary and secondary prevention activities can be effective at reducing IHD and related risk factors, although many reported modest and/or mixed results. The paucity of studies in remote and very remote areas [16] particularly in light of the relatively high burden of IHD in these areas compared to metropolitan counterparts [25], shows this is an understudied population in Australia. This is consistent with findings from the US, where rural populations are also identified as disadvantaged, and understudied in terms of IHD burden and prevention [9]. Worldwide, there is a lack of published research on comprehensive interventions to reduce IHD, especially in rural populations [26], despite the recognition that prevention efforts at population level, aimed at modifiable risk factors, can be both cost-effective and sustainable approaches to reducing IHD burden in high risk communities [11, 26, 27]. A recent review by Papadakis and Moroz [11] of high quality international studies of IHD prevention efforts, found only one study focussed on rural areas [27], out of 15 included in the review.

All studies included in this review employed a non-randomized design, mostly without a control group (six of eight studies), and all were found to have a high risk of bias. Half of the studies did not provide clear details about the exact timeframe of the intervention period, and subsequent follow up, or plans to follow up. Lack of comprehensive follow-up has been identified as an issue with the quality of evidence around IHD prevention efforts in rural areas, internationally [26]. Research in rural areas is likely to be challenging due to limited resources, small population numbers, and geographical remoteness. The lack of eligible studies retrieved for this review may reflect either a true lack of action or a lack of research reporting and publishing of prevention programs operating in rural communities. It is possible that effective and targeted interventions are taking place in many rural settings; however, they are not being published in the academic literature for others to learn from. It is known that rural health professionals feel ill-equipped to undertake, complete, and publish research, and this is due to a lack of resources, supervision, and perceived skills in rural areas [28, 29]. Research with larger samples and more rigorous study designs are required to progress strategies for reducing IHD burden in rural Australia, and worldwide.

Relatively few of the studies included in this review, reported extensively on behaviour change outcomes. Those studies that did included behaviour measurements [17, 18, 20, 22–24], used self-reported data only and findings were mixed. Changes in smoking prevalence at follow up were reported by one study [20], but did not change. Changes in BMI or weight were reported in five studies [17, 18, 20, 23, 24], however all but one study [17] found significant differences from pre and post intervention. Heterogeneity among these studies also made it difficult to draw concrete conclusions on the effectiveness of these interventions in improving risk behaviours.

Reductions in BP through medication prescription and monitoring, were demonstrated in four of the five studies [17, 18, 20, 22, 23] that assessed BP as an outcome, suggesting that perhaps, if similar interventions were implemented more broadly in rural areas, significant population-level impacts on blood pressure levels could be achieved. A reduction of 5 mmHg in diastolic BP level has been suggested to reduce population mortality from IHD by approximately 21 % [30, 31], and BP reduction is widely accepted as an important target risk factor, along with smoking and cholesterol levels, when aiming to prevent IHD in high risk populations internationally [7, 9, 30].

The results of this review showed a strong emphasis on clinical measurements rather than behavioural measures as outcomes when evaluating IHD prevention efforts. This pattern is evident in other international research, including a large scale intervention in rural Maine in the US, that was spread across 23 rural communities from 1970 to 2010 [27]. Although the intervention consisted of nurse delivered education focussed on behaviour changes, prescription medication and monitoring, cholesterol and BP were the main measured outcomes [18, 22]. This study provided some evidence that nurse-led education programs can reduce IHD risk factors in rural communities, but again, did not compare to a control group [27]. The focus on clinical risk factors as outcomes, as opposed to behavioural changes may be due to the possible bias that can arise from these self-reported measures, with clinical risk factor measures providing more concrete evidence of the effectiveness of the IHD prevention programs.

Higginbotham et al. [19] identified challenges with implementing a whole community intervention, and noted resistance when trying to engage the whole community, finding that only 35 % of people in the region thought IHD was of high concern [19]. Conclusions from this study included that when designing interventions for rural communities, use of existing structures and knowledge of the needs and interests of local subgroups is fundamentally important [19]. The authors of studies in the US and UK have also emphasized the importance of early assessment of rural community needs, interests and subgroups prior to implementation, when focussing on reducing IHD risk factors [7, 9, 32]. Conclusions from a review of international population-level interventions to reduce IHD showed that there is no ‘one size fits all’ program, and success of interventions relies heavily on due consideration of the needs, interests, characteristics and location of the community [11]. This is certainly applicable to the Australian rural context, which comprises of many diverse and different communities across a very large landscape.

Notable from this review was that only two of the selected studies were published in the past 5 years, with the remainder being eight or more years old, possibly limiting the relevance of these studies to when considering the current rural health context. The age of the evidence may be of concern given that Australia has seen both significant changes in the burden of IHD, and substantial shifts in urbanisation and population characteristics over recent decades [33]. Further, advances in technology, including e-health and telecommunications, may alter the experience and health implications of living in a non-metropolitan area. Therefore, rural populations presented in older studies, and the issues they faced, may not be comparable to those currently residing in rural areas.

Strengths of this research include that it employed a systematic methodology with broad search terms and included all Australian-based studies from 1990 to 2015. The comprehensive search strategy and use of three researchers to screen results increased the chances of all appropriate results being identified and included. The limitations of this review are that there was also only a small sample of studies that met the inclusion criteria, which had high heterogeneity and risk of bias in the methods, and this made solid conclusions on the effectiveness of interventions in rural areas difficult to decipher. The review was also limited to Australian studies only. While there are important similarities between rural populations across comparable countries, rural populations may differ significantly in demographic characteristics and disadvantage between different countries [34].

### Implications for future research

A major finding from this review is the lack of high quality studies assessing the effectiveness of interventions to reduce IHD burden in rural areas of Australia. Priority must be placed on action-orientated prevention research in rural communities, with a focus on methodologies such as community-based participatory research to empower communities, create tailored strategies and address inequalities [35] Future research must have specific focus on rigorous methodology, including comparison to control groups, more comprehensive measurements of changes in modifiable risk factors, and longer follow-up time frames in order to assess sustainability of such programs in the rural context. . There is significant potential for countries with comparable rural health inequalities (for example, geographically large, high income countries such as Australia, Canada and the US) to learn from international examples [7, 9, 26, 27] and create a stronger body of evidence for the prevention of IHD among rural populations globally international examples and create a stronger body of evidence for the prevention of IHD among rural populations globally.

## Conclusions

There are very few studies on interventions to prevent and reduce IHD in regional and remote areas of Australia, despite the higher burden in rural areas being well documented and this is consistent with international observations. Published interventions have generally shown encouraging results in reducing IHD risk factors or outcomes, although there significant limitations to quality and external validty among the studies identified. More research is needed to determine appropriate, feasible, effective, and cost-effective strategies for improving IHD rates in rural areas, and the best ways to tailor interventions to the specific needs of rural communities.

## Additional files

Additional file 1: PRISMA checklist. (DOC 62 kb) Additional file 2: Planned search terms. (DOCX 22 kb)

## Abbreviations

ATSI: Aboriginal and Torres Strait Islander
CVD: Cardiovascular disease
IHD: Ischaemic heart disease
NCDs: Non-communicable diseases
95 % CI: 95 % confidence interval
