# Supplementary material for: A systematic review of published interventions for primary and secondary prevention of ischaemic heart disease (IHD) in rural populations of Australia
Source: BMC Public Health. 2016 Aug 27;16(1):895. doi: 10.1186/s12889-016-3548-1 (PMC5002213; doi:10.1186/s12889-016-3548-1)
Supplement: Additional file 2: — Planned search terms. (DOCX 22 kb) [file 12889_2016_3548_MOESM2_ESM.docx]

**PLANNED SEARCH TERMS**

|  | **Concept 1** | **and**  **🡺** | **Concept 2** | **and**  **🡺** | **Concept 3** | **and**  **🡺** | **Concept 4** |
| --- | --- | --- | --- | --- | --- | --- | --- |
| **List all key words/terms as stated in your topic description above** | Cardiovascular  “Ischemic heart disease” |  | “Rural” |  | “Interventions” |  | Australia |
|  | **OR**  **🡻** |  | **OR**  **🡻** |  | **OR**  **🡻** |  |  |
| **List search terms using truncation* and wildcards?** | Coronary  Ischaem*  Ischem*  Heart  “Heart disease*” |  | Rural*  Region*  Remote*  Outside of major cities*  “Rural inequal*”  “Rural difference*” |  | Intervention*  Prevention* |  | Australia*  Victoria*  “New South Wales”  “Western Australia*”  “South Australia*”  “Northern Territory”  “Australian Capital Territory”  Queensland*  Tasmania* |
|  | **OR**  **🡻** |  | **OR**  **🡻** |  | **OR**  **🡻** |  |  |
| **List synonyms, alternate spelling, language, etc.** | Angina  “myocardial infarction”  “Cardiovascular outcome*”  AMI  IHD  CHD  Cardiac* |  | Country  Country Areas  Farm* |  | Program*  Change*  Trial*  Crossover  Qausi-experimental |  |  |
|  | **OR**  **🡻** |  | **OR**  **🡻** |  | **OR**  **🡻** |  |  |
| **List words derived from available thesaurus** |  |  |  |  |  |  |  |

**Databases:**

1. **CINAHL**
2. **Medline**
3. **Academic Search complete**
4. **Rural and remote health database**
5. **Health and society database**
6. **Embase**
